# A reassessment of the phylogenetic utility of genus-level morphological characters in the family Bogidiellidae (Crustacea, Amphipoda), with description of a new species of *Eobogidiella* Karaman, 1981

**DOI:** 10.3897/zookeys.610.9100

**Published:** 2016-08-11

**Authors:** Dmitry A. Sidorov, Aron D. Katz, Steven J. Taylor, Mikhail V. Chertoprud

**Affiliations:** 1 Institute of Biology and Soil Science, Far Eastern Branch of the Russian Academy of Sciences, 100-let Vladivostoku Av. 159, Vladivostok 690022, Russia; 2 Illinois Natural History Survey, University of Illinois, 1816 S. Oak St., Champaign, Illinois, USA; 3 Department of Entomology, University of Illinois, 320 Morrill Hall, 505 South Goodwin Avenue, Urbana, IL 61801, USA; 4 Department of Hydrobiology, Moscow State University, Leninskie Gory 1/12, Moscow 119234, Russia

**Keywords:** Biodiversity, Subterranean fauna, Karnataka, Taxonomy, Phylogenetic analysis

## Abstract

Bogidiellidae is the most diverse and cosmopolitan family of stygobiotic amphipods, and inhabits a variety of subterranean biotopes, especially interstitial habitats. While the family is characterized by considerable sexual dimorphism, this dimorphism has adversely affected our understanding of the systematics of the group. Most species have restricted geographic ranges and occur in difficult to sample habitats, so it is common for individual species descriptions to be based on a single sex. In this work we revisit an analysis of morphological characters in an attempt to clarify their phylogenetic utility in resolving taxonomic relationships among genera by introducing a new species, two additional characters, and phylogenetic statistical support values. *Eobogidiella venkataramani* **sp. n.**, from a spring fed brook in the Shirawati River basin along the escarpment of the Western Ghats (Karnataka, India) differs from the only known congener, *Eobogidiella purmamarcensis*, from Argentina, in the structure of mouthparts, the shape and ornamentation on gnathopods and characters of the telson. Our phylogenetic analyses indicate that the available morphological characters are not sufficient to resolve phylogenetic relationships within Bogidiellidae, thus these characters alone cannot be used to determine the phylogenetic placement of *Eobogidiella venkataramani* **sp. n.** within the family. Nevertheless, *Eobogidiella venkataramani* **sp. n.** shares diagnostic characters with *Eobogidiella*, supporting placement of the new species in this genus. Our findings point towards a critical need to resolve relationships within the family using molecular approaches, along with the development of a suite of additional morphological characters for Bogidiellidae. This is the third species of Bogidiellidae from southern India.

## Introduction

The family Bogidiellidae Hertzog, 1936 has an intriguing history of study that shaped the systematics of the group (e.g., Hertzog 1933; Holsinger and Longley 1980; Karaman 1981; Stock 1981; Ruffo 1973; Koenemann et al. 1998; Koenemann and Holsinger 1999; Iannilli et al. 2006; Jaume et al. 2007; Vonk and Jaume 2010; Leijs et al. 2011; Senna et al. 2014), but this work has not led to a coherent understanding of relationships within the family (Lowry and Myers 2013). The Bogidiellidae includes 37 genera and 113 described species, with the phylogenetic relationships among the genera discussed by Stock (1981) and a phylogenetic tree produced by Koenemann and Holsinger (1999).

Only two Bogidiellidae species are known from India: *Bogidiella indica* Holsinger et al. 2006, recorded from bore wells in Andra Pradesh, and the minute species *Bogidiella totakura* Senna et al. 2013, from a nearby locality Andhra Pradesh, southern India. The only other stygobiotic amphipod species of India are the gammaroid *Indoniphargus indicus* (Chilton 1923) (Mesogammaridae), reported from various groundwater habitats (e.g., springs, well water, and a mine pit) in the north-eastern states of Bihar, West Bengal and Odisha (formerly Orissa) (Stephensen 1931; Straškraba 1967), and the crangonyctoid *Kotumsaria bastarensis* Messouli et al. 2007 (Kotumsaridae), from Kotumsar Cave, in the east-central state of Chhattisgarh (Messouli et al. 2007; Senna et al. 2013).

Below we describe *Eobogidiella venkataramani* sp. n. from a spring-fed freshwater habitat in southwest India and evaluate the phylogenetic utility of the available morphological characters (Koenemann and Holsinger 1999, and two characters added in the present study) in hopes of gaining insights into the placement of our new species within the family.

## Methods

### Specimen sampling

**Figure 1.**
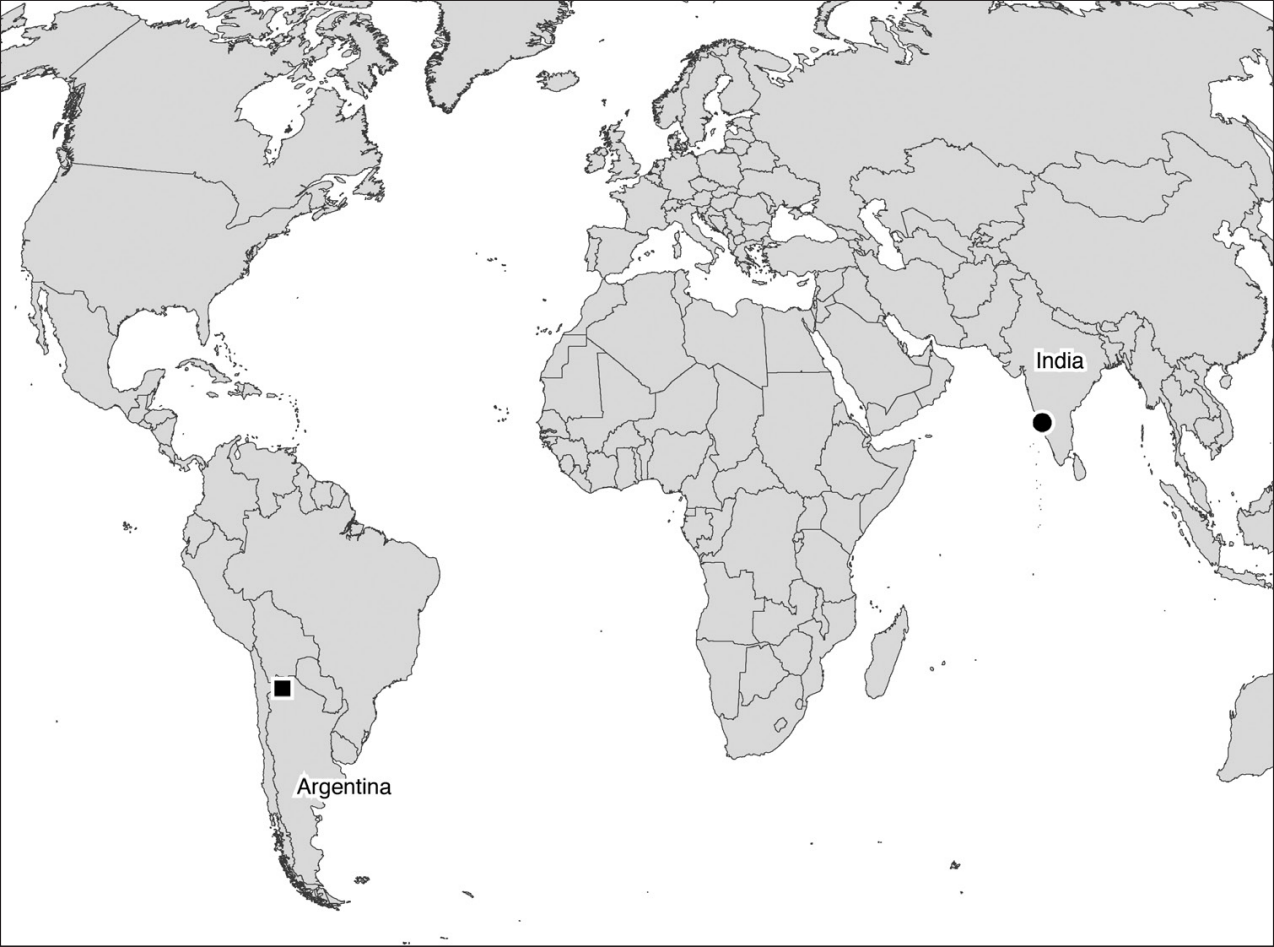
Geographic distribution of *Eobogidiella venkataramani* sp. n. (circle) and *Eobogidiella purmamarcensis* (Grosso & Ringuelet, 1979) (square).

**Figure 2.**
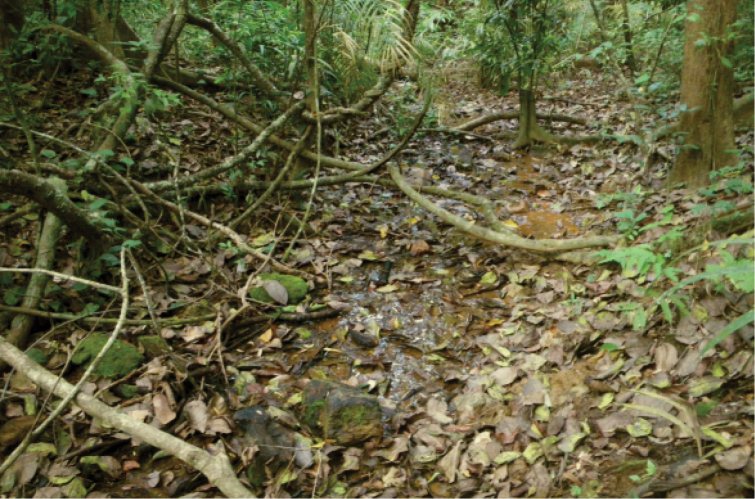
“Wet-spot” biotope in the Shirawati River basin, Western Ghats, India.

A sample containing the stygobiont (one specimen) was collected in December 2008 from a spring-fed brook in the state of Karnataka in southwest India (Figs 1, 2) using a hand-made hemispherical scraper and preserved in a 4% solution of formaldehyde.

### Morphology and taxonomic terms

Body length was recorded while holding the specimen straight and measuring the distance along the dorsal side of the body from the base of the first antenna to the base of the telson using an ocular micrometer in a Lomo MBS-9 dissecting microscope. Appendages were drawn using a Carl Zeiss NU-2 compound microscope equipped with a drawing device as described in Gorodkov (1961).

Due to improper storage, the specimen was entirely dry upon initial examination. We followed the method described by Namiotko et al. (2011) to rehydrate the specimen. A permanent preparation was made using polyvinyl lactophenol (PVL) and a methylene blue staining solution was used as mounting medium.

The term “palmar angle” of the gnathopod propodi refers to the angle formed at the end of the palm and beginning of the posterior margin or the point at which the tip of the dactylus closes on the propodus (Birstein 1941). The fore-gut lateralia comprise a potentially useful morphological character in the phylogenetic analysis (Coleman 1991). We use the term “sternal humps” (Holsinger 1989; Sidorov 2010) to refer to the “pulvinate sternal epithelium” of Kikuchi et al. (1993) and Kikuchi and Matsumasa (1997), which is homologous to the “mediosternal processes” of Koenemann and Holsinger (1999) and Pérez-Schultheiss (2013). Koenemann and Holsinger (1999) took the view that the mediosternal gills of *Paracrangonyx* Stebbing, 1899 are autapomorphous, with a different physiological function and morphological structure, but they do not cite the works of Kikuchi. Fenwick’s (2001) diagnoses of the genus *Paracrangonyx* includes the presences of “Single, simple, elongate sternal gills medially on peraeonites 2–7.” Bousfield (1977) observed that sternal gills are present in several families of amphipods which are not closely related – including Crangonyctidae (e.g., Holsinger 1977), Hyalellidae, and Pontogeneiidae – and suggests that these structures arose independently in the different groups. Homologies of mediosternal gills and sternal humps within and across families of freshwater amphipods remains problematic, and effective use of these characters in phylogenetic analyses requires further study.

### Phylogenetic analysis

To investigate the phylogenetic utility of the available morphological characters we used a revised version of the morphological data matrix used by Koenemann and Holsinger (1999) and incorporated phylogenetic methods that provide measures of statistical support (See Suppl. material 1 for morphological character matrix). Our updated data set includes additional genera described by various authors since Koenemann and Holsinger’s (1999) analyses (*Fidelidiella* Jaume, Gràcia & Boxshall, 2007; *Glyptogidiella* Vonk & Jaume, 2010; *Omangidiella* Iannilli, Holsinger, Ruffo & Vonk, 2006, *Racovella* Jaume, Gràcia & Boxshall, 2007), three additional species (*Patagongidiella wefkoi* Pérez-Schultheiss, 2013; *Xystriogidiella juliani* Coleman, 2009; *Eobogidiella venkataramani* sp. n.), two additional morphological characters, and published taxonomic updates made by Koenemann and Holsinger (1999) as follows: *Medigidiella* (was *Medigidiella* A), *Indogidiella* (was *Medigidiella* C), *Arganogidiella* (was *Medigidiella* B), *Bogidiella* (*niphargoides* group) (was *Bogidiella* C), *Bogidiella* (*skopljensis* group) (was *Bogidiella* B), *Bogidiella* (*albertimagni* group) (was *Bogidiella* A), *Stygogidiella* (was *Stygogidiella* A), and *Argentinogidiella* (was *Stygogidiella* B). The new morphological characters address the hypertrophied coxa 5 in *Glyptogidiella* and the unique position of the coxal gills on pereonite 7 in *Xystriogidiella juliani* (Coleman 2009). Iannilli et al. (2005) discussed another character, “coxal endite on maxilliped (or third coxal lobe)”. We were unable to code the coxal endite character of Iannilli et al. (2005) for most genera, as this character is not included in earlier descriptions.

We used the Bogidiellidae *sensu lato* in our analysis, including Artesiidae, as its acceptance as a distinct family has been questioned (Stock 1981; Botosaneanu and Stock 1989), as well as the genus *Kergueleniola* Ruffo, 1974 which is sometimes placed in a separate family Kergueleniolidae (Lowry and Myers 2013). We were unable to test the validity of the inclusion of the Salentinellidae in Bogidielloidea within the Senticaudata: the uniramous uropod 3 in *Parasalentinella* Bou, 1971 does not fit with core bogidiellid features, and *Salentinella* Ruffo, 1947 species lack apical robust setae on uropods 1–2 (cf., *Salentinella anae* Messouli et al. 2002). *Bogidiella indica* Holsinger (2006), the sole member of the *indica*-group *sensu* Holsinger et al. (2006), recently has been attributed to the *niphargoides*-group based on the shared absence of rami on pleopods 1–3 (Senna et al. 2013). Therefore, the *indica*-group was not considered in our analysis, as it is instead included in our analysis within the *niphargodes*-group. The genera *Paracrangonyx* Stebbing, 1899 (Paracrangonyctidae), *Pseudingolfiella* Noodt, 1965 (Pseudingolfiellidae) and *Dussartiella* Ruffo, 1979 (Dussartiellidae) were excluded from the analysis. The recent placement of these genera in different families (see Koenemann and Holsinger 1999; Iannilli et al. 2011; Lowry and Myers 2012), supports a higher-level analysis of the Senticaudata, in which the Pseudingolfiellidae is not considered even to be a member of the suborder Senticaudata (Lowry and Myers 2013), whereas the Paracrangonyctidae and Dussartiellidae fall into the Gammarida instead of the Bogidiellida in the analysis of Lowry and Myers (2013).

Following Koenemann and Holsinger (1999), we conducted two phylogenetic analyses, treating all characters as unweighted: first with unordered character states and an ‘alternative’ analysis with ordered character states. The parsimony analyses (unordered and ordered) of 46 taxa, including 37 genera of Bogidiellidae, 2 genera of Artesiidae, and 1 genus of Kergueleniolidae, and the hypothetical ancestor outgroup used by Koenemann and Holsinger (1999), were based on 29 morphological characters (Suppl. material 1). Both analyses were conducted in PAUP*4.0a146 (Swofford 2002) using a heuristic search, random stepwise addition with 1000 replicates and TBR branch swapping. Advances in computer power and processor speeds and have allowed us to reevaluate Koenemann and Holsinger’s (1999) original cladistic analysis with modern and more rigorous methods that incorporate statistical measures of branch support. Bootstrap and Jackknife resampling methods for branch support were performed with PAUP*4.0a146 using the “Fast” stepwise-addition search (1,000,000 replicates). PAUP* command files for Decay/Bremer support indices were generated with TreeRot. v3 (Sorenson and Franzosa 2007), input with strict consensus trees, edited to run each heuristic search for 500 replicates with TBR branch swapping, and executed in PAUP*4.0a146.

### Acronym used for the collection

FEFU
 Zoological Museum of the Far East Federal University, Vladivostok 


## Results

### Phylogenetic analysis of Bogidiellidae *sensu lato*

**Figure 3.**
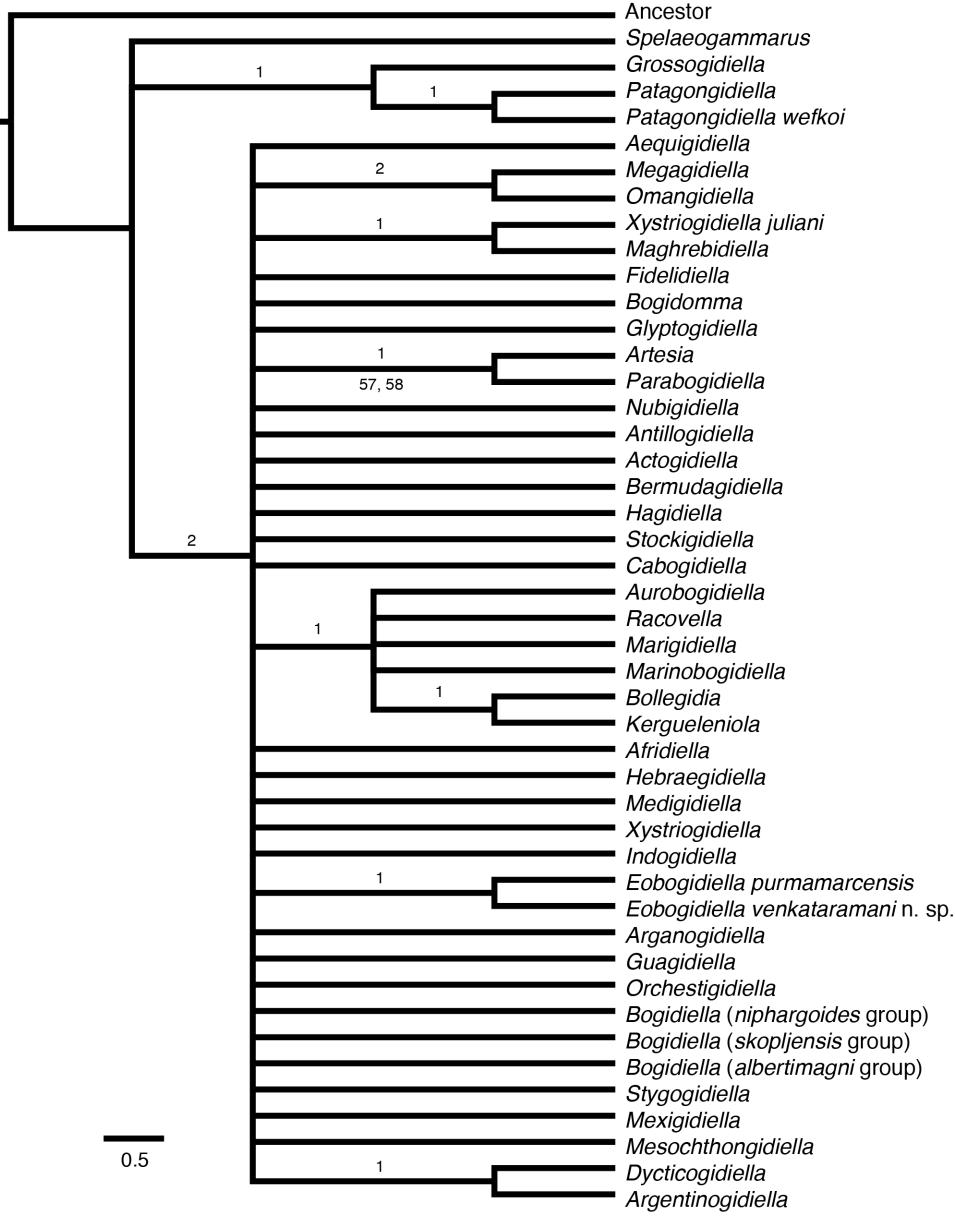
Maximum parsimony strict consensus tree of genera and selected species of Bogidiellidae, ordered analysis. Numbers above branches are Decay/Bremer indices and numbers below branches are bootstrap followed by jackknife support values. Support values less than 50% not displayed. Scale bars indicate number of character state changes. See Suppl. material 2 for original bootstrap and jackknife consensus trees.

**Figure 4.**
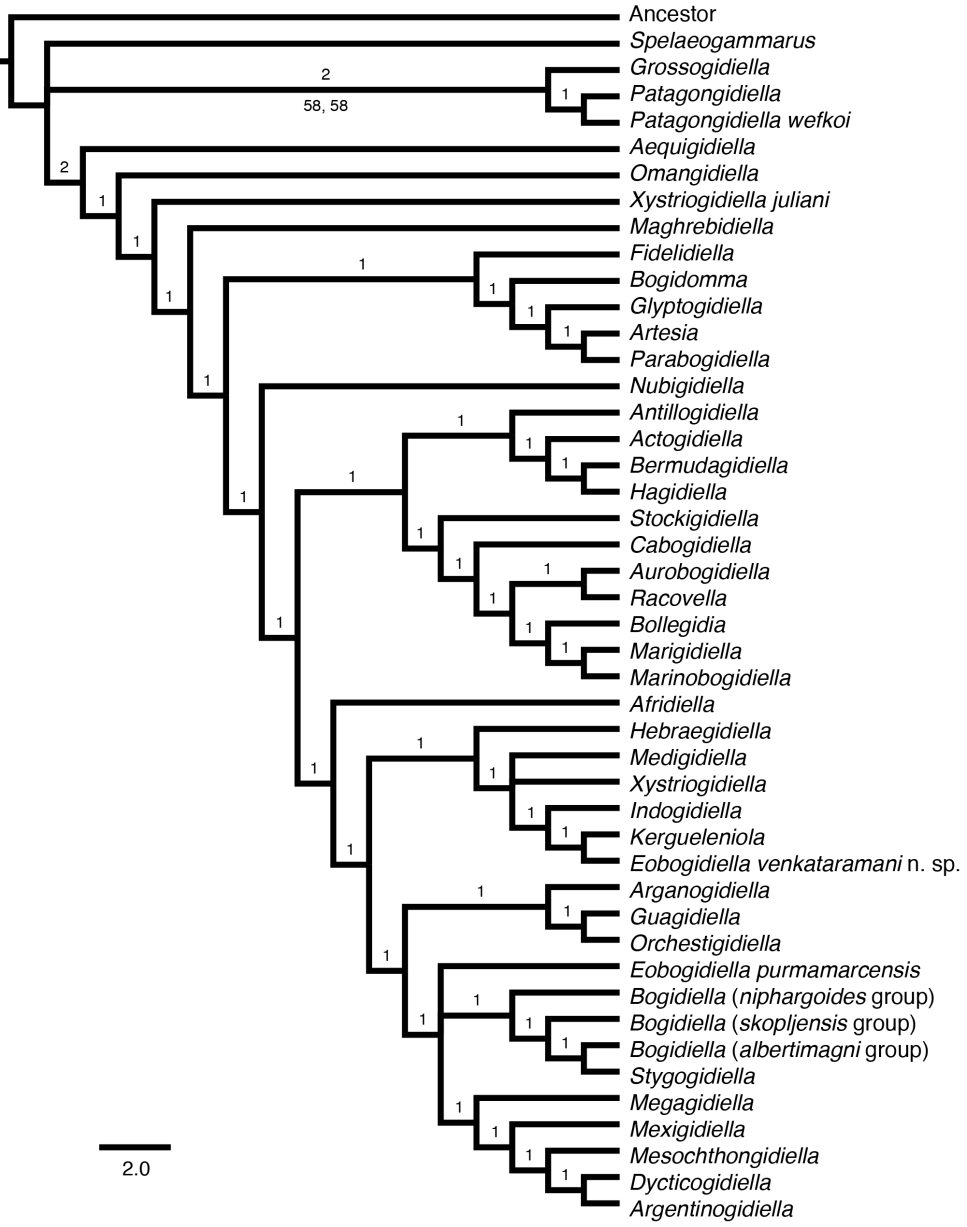
Maximum parsimony strict consensus tree of genera and selected species of Bogidiellidae, unordered analysis. Numbers above branches are Decay/Bremer indices and numbers below branches are bootstrap followed by jackknife support values. Support values less than 50% not displayed. Scale bars indicate number of character state changes. See Suppl. material 3 for original bootstrap and jackknife consensus trees.

To investigate the phylogenetic utility of the available morphological characters and to determine the placement of our new species among the bogidiellids, we reevaluated the relationships within the family, adding new taxa and characters to the morphology matrix of Koenemann and Holsinger (1999). Our phylogenetic analysis of 29 morphological characters supports two equally parsimonious trees (length = 170) and 3235 equally parsimonious trees (length = 243) in the unordered and ordered analyses, respectively. Although the strict consensus trees (Figs 3, 4) resolved some relationships, they lack support from bootstrap (Suppl. material 2, 3), jackknife (Suppl. material 2, 3), and Bremer/decay indices (Figs 3, 4), due, at least in part, to the low character to taxa ratio (29 to 46, respectively). The strict consensus tree for the ordered analysis places the new species within the genus *Eobogidiella* Karaman, 1981 (Fig. 3), without significant statistical support. The unordered analysis (Fig. 4) instead places these two taxa in association with other genera (the new species with *Kergueleniola*; *Eobogidiella purmamarcensis* (Grosso & Ringuelet, 1979) with *Bogidiella* and other genera), also without significant statistical support.

The two additional characters (i.e., the presence or absence of a coxal endite on the maxilliped, and the morphology of coxa 5) were added to the matrix of Koenemann and Holsinger (1999), but provide little additional phylogenetic support. Although the “maxilliped, coxal endite” is an informative character as it is present for a number of genera, the morphology of coxa 5 is normal for all genera except for *Glyptogidiella* (for which it is hypertrophied), therefore, this autapomorphy is phylogenetically uninformative.

The above analyses revealed that the available morphological characters provide no phylogenetic utility in resolving generic relationships within the Bogidiellidae *sensu lato*, thus the available morphological characters do not allow us to establish the phylogenetic placement of the new species. Therefore, the resulting phylogenies (Figs 3, 4) cannot be used to inform generic placement of the new species or direct choices for generic comparisons. Instead, generic placement of the new species must rely exclusively on shared generic-level diagnostic characters. Generic-level diagnostic characters (i.e., 3 outer ramus segments in pleopods 1–3, uniarticulate and reduced inner rami of pleopods 1–3, and 1 segmented palp of maxilla 1) of the new species are shared with the South American genus *Eobogidiella*, suggesting a possible close relationship with *Eobogidiella purmamarcensis*. Other bogidiellid genera were considered based on the shared presence and absence of male sexual modifications (*Indogidiella*) and similar geographical distributions (*Bogidiella*) (Table 1). Although both *Indogidiella* and the new species lack modifications of the outer ramus in male pleopods 1 and 2 and have modified spines on rami of male uropods 1 and 2 (Table 1), the nature of the modifications of the spines on the rami of male uropods 1 and 2 of *Indogidiella* (Ruffo 1994, fig. 4g, h; Stock 1983, figs 23, 24) differ from the new species, possibly indicating independent origins. Furthermore, species in *Indogidiella* have a 2 segmented palp on maxilla 1, whereas the new species has a 1 segmented palp. The geographically proximate species, *Bogidiella indica* and *Bogidiella totakura*, do not share the same male sexual modifications and also have a 2 segmented palp on maxilla 1. Therefore, based on the diagnostic characters shared with *Eobogidiella* and morphological dissimilarity from *Indogidiella* and the more geographically proximate genus, *Bogidiella*, we tentatively place the new species in the genus *Eobogidiella* recognizing further study is required to understand generic boundaries and relationships within the family.

**Table 1.** Species distributions and selected morphological characters from for *Eobogidiella purmamarcensis*, *Indogidiella daccordii*, *Indogidiella sarawacensis*, *Bogidiella indica*, *Bogidiella totakura*, and the new species. Characters listed in the table represent all morphological characters from Koenemann & Holsinger (1999) that are variable among presented taxa. Bold character states indicate that the state is shared with the new species.

Characters	*Eobogidiella venkataramani* sp. n.	*Eobogidiella purmamarcensis* (Grosso & Ringuelet, 1979)	*Indogidiella daccordii* (Ruffo, 1994)	*Indogidiella sarawacensis* (Stock, 1983)	*Bogidiella indica* Holsinger et al., 2006	*Bogidiella totakura* Senna et al., 2013
Distribution	India	Argentina	Phillipines	Borneo	**India**	**India**
Modifications of the outer ramus in male pleopod 1	absent	?	**absent**	**absent**	**absent**	?
Modifications of the outer ramus in male pleopod 2	absent	?	**absent**	**absent**	**absent**	?
Number of outer ramus segments in pleopods 1-3	3	**3**	**3**	**3**	**3**	**3 or 4**
Inner rami of pleopods 1-3	uniarticulate, reduced	**uniarticulate, reduced**	**uniarticulate, reduced**	**uniarticulate, reduced**	absent	absent
Modifications in male uropod 1	present	?	**present**	**present**	**present**	?
Modifications in male uropod 2	present	?	**present**	**present**	**absent**	?
Dagger-shaped rami in male uropod 1	absent	?	**absent**	**absent**	**absent**	?
Dagger-shaped rami in male uropod 2	absent	?	**absent**	**absent**	**absent**	?
Gills	pleopods 3-6	?	?	pleopods 4-6	pleopods 2-6	pleopods 4-6
Number of segments in flagellum of antenna 2	5	**5**	**5**	6	**5**	**5**
Number of segments in accessory flagellum	1	2	3	3	**1**	3
Number of palp segments in maxilla 1	1	**1**	2	2	2	2
Number of setae on inner lobe of maxilla 1	2	3	**2**	**2**	4	0
Number of spines on outer lobe of maxilla 1	7	**7**	**7**	**7**	6	6
Mandibular molar	non-triturative	triturative	triturative	triturative	**non-triturative**	“semi-triturative”
Number of apical spines of telson	0	1	1	2	1	**0**
Number of subapical spines of telson	2	3	0	0	0	1

### Species description and taxonomy Order Amphipoda Latreille, 1816 Family Bogidiellidae Hertzog, 1936

#### Genus Eobogidiella

Taxon classificationAnimaliaAmphipodaBogidiellidae

G. Karaman, 1981

Bogidiella (Eobogidiella) G. Karaman 1981: 34, syn. —Eobogidiella G. Karaman 1982: 50. —Koenemann and Holsinger 1999: 797, 810. —Lowry and Myers 2012: 43. —Mexigidiella (part.) Stock 1981: 354.

##### Type species of the genus.

Bogidiella (Eobogidiella) purmamarcensis Grosso & Ringuelet, 1979, (by original designation).

#### Eobogidiella venkataramani sp. n.

Taxon classificationAnimaliaAmphipodaBogidiellidae

http://zoobank.org/B0EE2445-3C65-45D0-B7C6-8ECF6ECE701A

Figs 5 , 6 , 7

##### Diagnosis.

Habitus typical of a stygomorphic bogidiellid, combining a number of features found in other genera of this family.

Primary characters: maxilla 1 with vestigial, single-segmented, symmetrical palps; pleopods 1–3 with single-segmented, reduced inner rami.

Secondary characteristics: ventral surface of pereonites 2–7 bearing sternal humps; coxal gills on pereopods 3–6; antenna 1 with reduced, single-segmented, minute accessory flagellum; mandibles with tiny, vestigial molars with 2 short spines and 1 plumose seta; maxilliped lacking coxal endite; apparent sexual dimorphism (spines on uropods 1 and 2 modified).

##### Type locality.

Spring fed swamp in the upper reaches of a small logged brook (14.218667°N; 74.821667°E) in the Shirawati River basin, altitude above sea level 550 m, Western Ghats, Karnataka, India.

##### Type material.

**Holotype specimen.** INDIA: probable ♂, 6.5 mm, X43794/Cr-1621-FEFU, vicinity of Jog Falls, Karnataka state, collected 5 Dec. 2008 by M.V. Chertoprud. Deposited in the Zoological Museum of the Far East Federal University, Vladivostok (FEFU).

Accompanying fauna: *Goerodes* sp. (Trichoptera: Lepidostomatidae), *Isca* sp. (Ephemeroptera: Leptophlebiidae), *Phanoperla* sp. (Plecoptera: Perlidae), *Macromyia* sp. (Odonata: Corduliidae), and many terrestrial leeches (Hirudinida) on the banks.

##### Etymology.

The specific epithet honors the former Director of Zoological Survey of India, Dr. K. Venkataraman, whose assistance was pivotal in the early stages of this research.

##### Description of holotype

**Figure 5.**
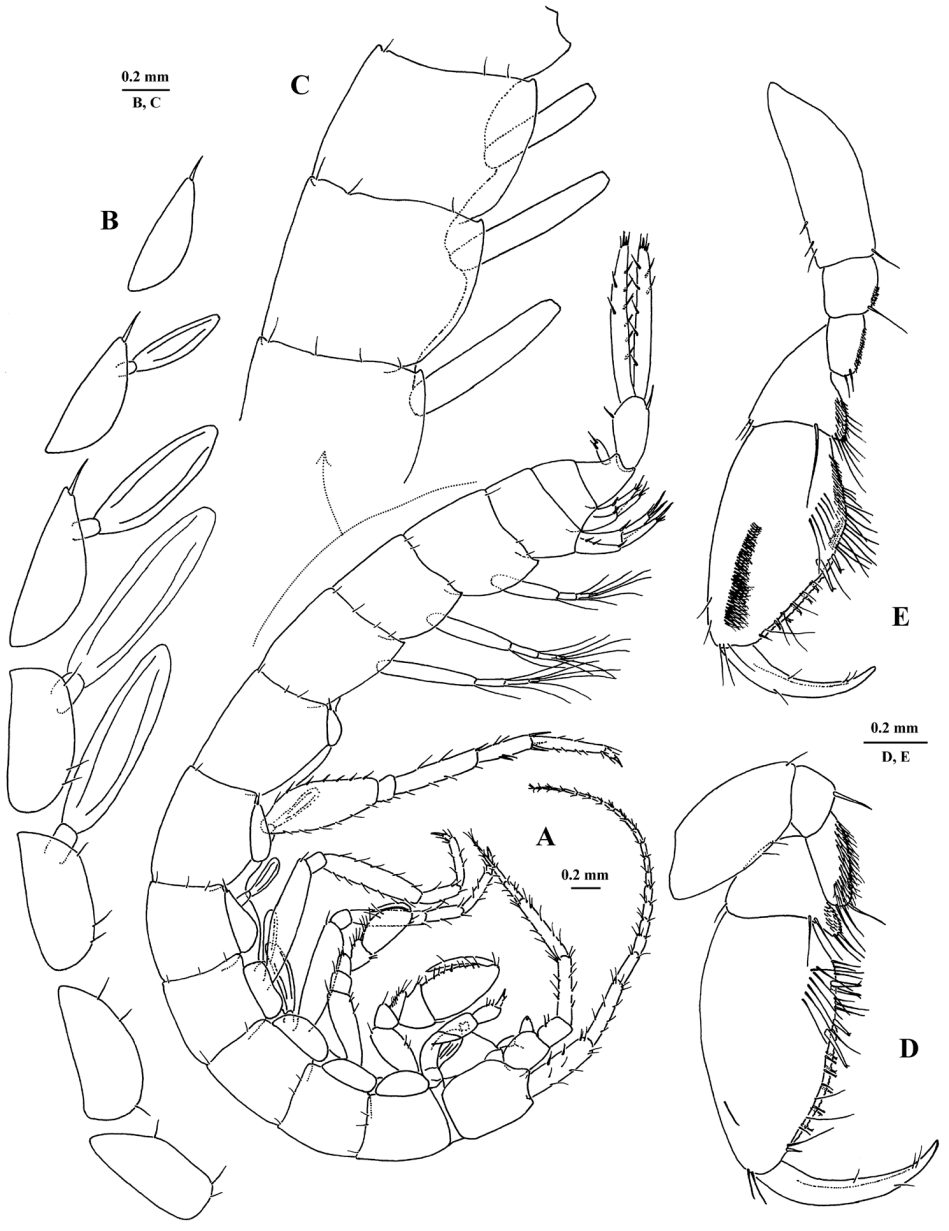
*Eobogidiella venkataramani* sp. n., ♂ (?), 6.5 mm, holotype, Jog Falls, Karnataka, India: **A** habitus from left side **B** coxae 1–7 **C** epimeral plates 1–3 **D, E** gnathopods 1–2.

**Figure 6.**
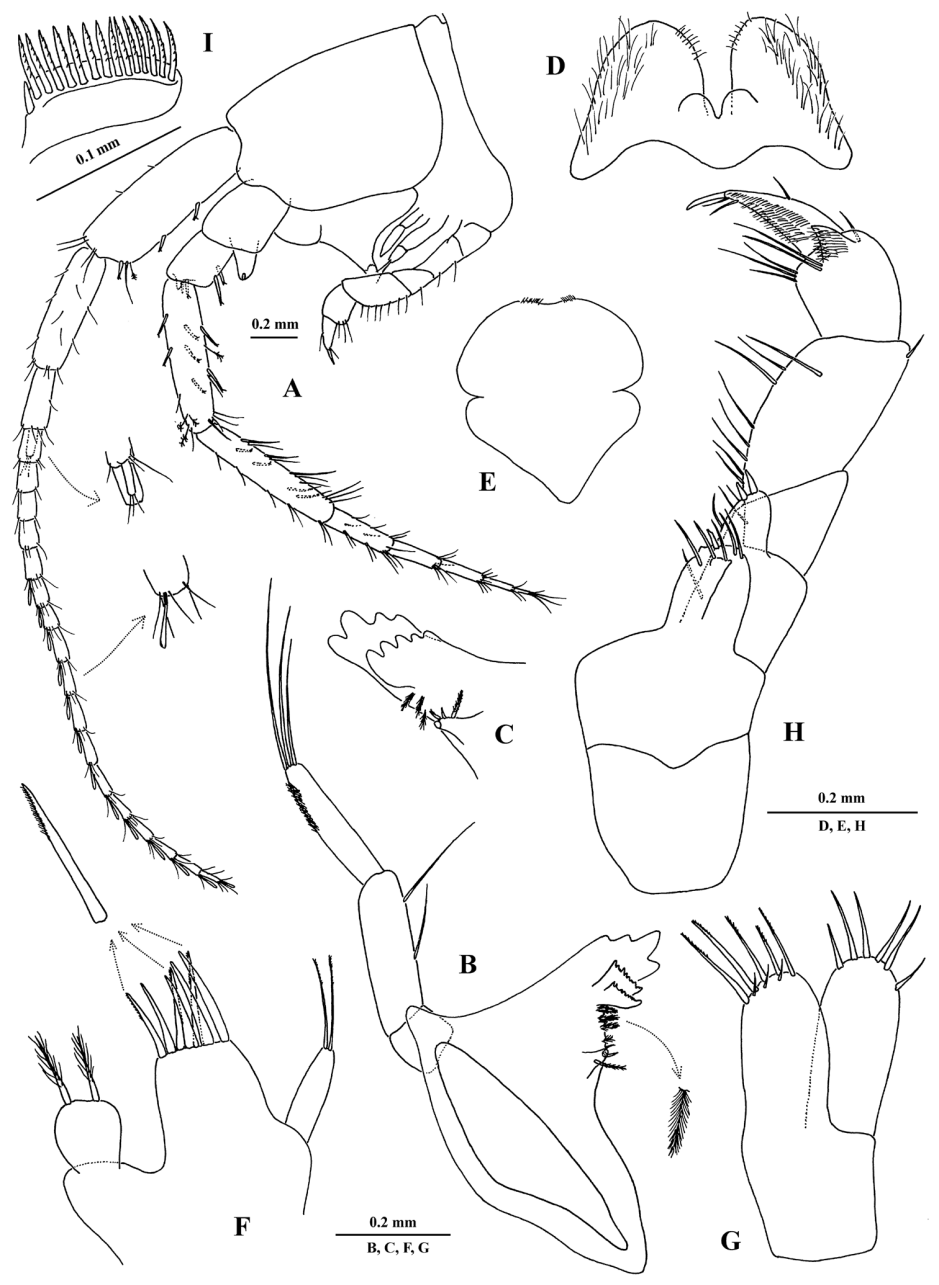
*Eobogidiella venkataramani* sp. n., ♂ (?), 6.5 mm, holotype, Jog Falls, Karnataka, India: **A** head **B** left mandible **C** incisor and lacinia mobilis of right mandible **D** labium **E** labrum **F, G** maxillae 1–2 **H** maxilliped **I** lateralia.

**Figure 7.**
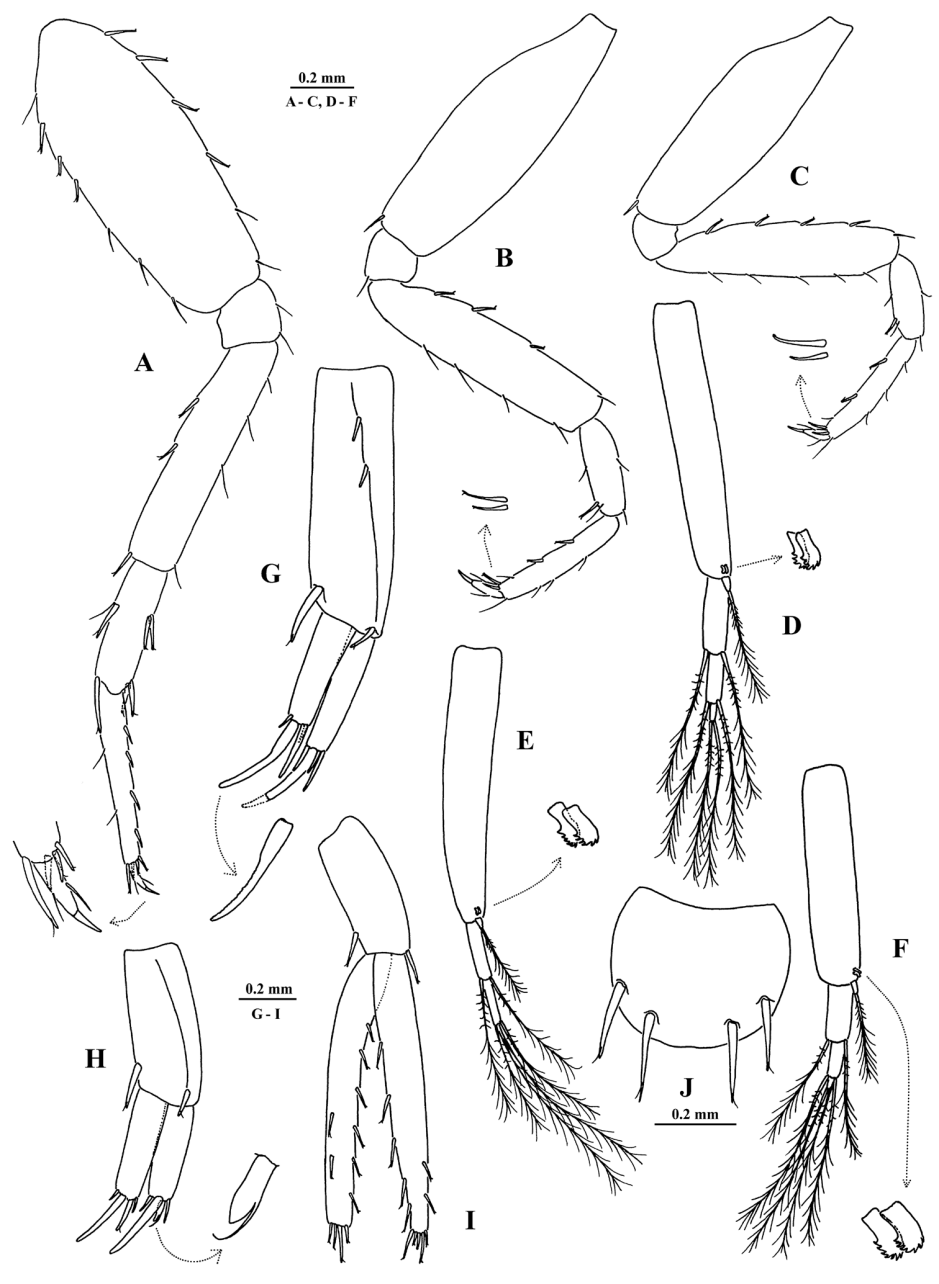
*Eobogidiella venkataramani* sp. n., ♂ (?), 6.5 mm, holotype, Jog Falls, Karnataka, India: **A** pereopod 6 **B, C** pereopod 3–4 **D, E, F** pleopods 1–3 **G, H, I** uropods 1–3 **J** telson. Pereopods 5 and 7 are missing.

**X43794/Cr-1621-FEFU.** General body morphology (Figs 5A, C, 6A, 7J). Body unpigmented, smooth, sparsely setose with fine setae. *Head* longer than deep and longer than first pereon segment; rostrum pointed, interantennal lobe distinct, evenly rounded apically; eyes absent. *Epimeral plates 1–3* with acute posterodistal corners and with thin setae on posterior margin, ventral margin of plates unarmed. *Telson* subquadrate with apical margin roundly convex, width: length ratio 1 : 0.75, bearing 4 long notched spines subapically. ANTENNAE (Figs 5A, 6A). *Antenna 1* about 38% of body length; flagellum with 17 articles, each article with 2–4 short setae, aesthetascs present on 12 distal flagellar articles; peduncular article ratio 1 : 0.67 : 0.3; proximal article of peduncle with 3 notched spines on ventral margin; accessory flagellum small, comprised of one article. Ratio of lengths of antenna 1 : antenna 2, 1 : 0.75; flagellum of *antenna 2* with 5 articles, each article sparsely setose; peduncle article 4 as long as article 5; flagellum shorter than peduncle (articles 4+5); last two peduncular articles with notched spines and long, stiff setae; gland cone not markedly elongate. MOUTH PARTS (Fig. 6A–I). *Labrum* subtrapezoidal, long as broad, clypeus unfused. Inner lobes of *labium* well developed, outer lobes broad, densely setose laterally, with thin setae marginally, and lightly setose with shorter setae mediodistally, mandibular process narrow. *Left mandible*: incisor with 4 teeth, lacinia mobilis consisting of 2 finely denticulate plates of similar size; row of 3 densely plumose spines between lacinia and molar; molar vestigial, conical, bearing 2 short spines and 1 plumose seta. *Right mandible*: incisor with 4 teeth, lacinia mobilis with 5 teeth, row of 2 densely plumose spines between lacinia and molar; molar similar to that of left mandible. *Mandibular palp* article 2 slightly longer and broader than article 3; proximal palp article without a seta; the second article with 2 long setae on inner margin; distal article narrow, with 3 long setae unequal in length on apex, and numerous small, fine setae near lateral margin on distal half of article. *Maxilla 1* palp reduced, single-segmented, with 2 long setae of equal length on apex (palps symmetrical); outer plate with 7 simple spines, 3 of which are finely pectinate; inner plate broadly rounded distally, with 2 plumose setae. *Maxilla 2* plates similar in size, inner plate with 6 apical setae of varying size, outer plate with 5 long, finely pectinate setae and 3 short setae apically. *Maxilliped* with inner and outer plates short; outer plate with 2 apical spines accompanied by 2 stiff setae on lateral face; inner plate broad, with 1 bifid apical spine and 3 stiff naked subapical setae, 2 setae located medially on small pedestal; palp four-segmented; palp article 2 longest, nearly straight on outer margin, shallowly convex on inner margin, with a row of 8 long, simple setae along inner margin; article 3 half as long as article 2, with sharply pointed, pubescent cuticular projection distally and bearing 2 sets of long setae apically; article 4 about as long as preceding article, curved and tapering distally, with dorsal seta, and bearing 2 longer setae at base of nail, nail 0.33× length of pedestal. *Lateralia* with 14 strong, pectinate spines and 1 short simple spine. COXAL PLATES, GILLS AND STERNAL RESPIRATORY STRUCTURES (Fig. 5A, B). *Coxal plates 1–7* wider than long, free, not overlapping with one another, coxa 4 largest; *coxal plates 5–7* progressively smaller towards the posterior, semicircular, acuminate posteriorly and bearing 1 stiff seta posteriorly. *Coxal gills* oblong, stalked on coxae 3 to 6. Ventral surface of pereonites 2–7 bearing *sternal humps*. GNATHOPODS 1 AND 2 (Fig. 5D, E). *Gnathopod 1*, basis short, broadest medially, with 2 short setae on anterior margin; merus with 3 stiff setae on distoposterior margin, posterior surface densely spinose; carpus sub-triangular, with 2 setae of equal length on narrowly rounded spinose distoposterior lobe; propodus oblong, about 1.8× longer than broad, palmar margin slightly convex, 3× longer than posterior margin, palmar angle indistinct, with 1 group of oblique, long setae laterally on basal half of segment; anterior margin with 1 seta, and a group of 2 setae anterodistally; palm armed with 2 pairs of weakly notched spines accompanied by 10–12 stiff, tiny notched setae along inner and outer faces; dactylus falcate, about 70% length of propodus, demarcation of nail indistinct with 2 setules at hinge. *Gnathopod 2*, basis sublinear, with 3 short setae on distal one third of anterior margin; ischium posterior surface densely spinulose with one longer, posterodistal seta; merus with posterior surface densely spinulose, with two stiff longer, posterodistal seta; carpus triangular and slightly elongate, with numerous thin subequal setae on broadened, spinulose ventral lobe, 1 long seta distally on medial face; propodus small, slightly shorter than propodus of gnathopod 1; palmar margin oblique, subequal in length to posterior margin, palmar angle poorly developed and broadly rounded, with 1 group of oblique long setae subdistally; anterior margin with 2 setae, anterodistal group with 3 setae; palm armed with 1 pair of weakly notched spines accompanied with 5–6 stiff, tiny, notched setae along inner and outer faces; dactylus similar to that of gnathopod 1. PEREOPODS 3, 4, 6 (pereopods 5 and 7 missing) (Fig. 7A–C); lacking lenticular organs. *Pereopods 3–4* subequal, bases rather long and narrow, each with 1 stiff seta on anterodistal margin; dactyli about 0.33× length of corresponding propodi. *Pereopod 6* length 0.35× body length; basis narrowed distally, length:width is 1:0.4; posterior margin with 3 notched spines and 4 setae; anteriorly 4 notched spines and 2 setae; carpus short, length 0.5× preceding article, armed with strong spines on lateral and distal margins; dactylus about 0.25× length of corresponding propodus. PLEOPODS AND UROPODS (Fig. 7D–I). *Pleopods 1–3* subequal; peduncular articles linear, in ratio 1:1:0.7, with 2 retinacula each; inner ramus reduced, 1-segemented, length less than basal width of first segment of outer ramus; outer ramus 3-segmented, fringed with long, plumose setae at distal end of each segment. *Uropod 1* peduncle without basofacial spine; with 3 dorsolateral spines and distally with 1 very strong dorsomedial spine; exopodite:endopodite length 1:0.88; endopodite length 0.5× peduncle; rami straight, each armed with 4 strong spines apically, 1 of them much larger and with marginal serrations. *Uropod 2* peduncle with 1 dorsolateral spine and 1 strong dorsomedial spine distally; exopodite:endopodite length 0.86:1; endopodite length 0.7× peduncle; rami straight, each armed with 4 spines apically, 1 of them much larger and another modified (Fig. 7H). *Uropod 3* long, with peduncle about 1 half the length of rami, armed with two notched spines on apex; endopodite curved in basal half, with 8 singly inserted notched spines along margins and 4 apical spines; exopodite straight, slightly tapering in distal half, with 6 singly inserted notched spines along margins and 5 apical spines.

##### Variability.

Unknown.

##### Sexual dimorphism.

Unknown, but modified spines on uropods 1 and 2 probably represent a male-specific trait.

##### Distribution and ecology.

*Eobogidiella venkataramani* sp. n. dwells in a spring-fed brook habitat located on the flat bottom of a small valley in the rainforest. The biotope is a small trickling swampy stream 1–3 m wide and 0–0.05 m deep, without flow, water temperature +22 °C, and a substrate comprised of wet litter, detritus, stones, clay. Known only from type locality.

##### Taxonomic comments.

*Eobogidiella venkataramani* sp. n. is distinguished from *Eobogidiella purmamarcensis* by the following characteristics (characteristics of the latter in parentheses): antenna 2 reaching 75% of antenna 1 length (about 50%); accessory flagellum comprised of 1 article (2 articles); molar vestigial, non-triturative (developed, triturative); mandibular palp article 3 with 3 setae on apex (1 seta); maxilla 1 inner plate with 2 setae (3 setae); maxilla 2 plates broad (narrow); maxilliped palp article 2 narrow (very broad); lenticular organs absent (present); telson with apical margin convex (with excavation apically).

## Discussion

The only other species in this genus, *Eobogidiella purmamarcensis* was described by Grosso and Ringuelet (1979) who placed it in the genus *Bogidiella*. It occurs in sandy sediments of the Rio Grande at the entrance of Purmamarca, Jujuy Province of northwestern Argentina (Fig. 1). Karaman (1981) places this species, along with *Marigidiella brasiliensis* (Siewing, 1953) (formerly *Bogidiella brasiliensis*), in the Bogidiella subgenus Eobogidiella. At about the same time, Stock (1981) tentatively attributed *Bogidiella purmamarcensis* to the subgenus Mexigidiella whereas *Bogidiella brasiliensis* removed to the new genus *Marigidiella*. A year later, Karaman (1982) elevated *Eobogidiella* to generic status.

In spite of our decision assign the new species to *Eobogidiella*, weak phylogenetic support for generic concepts and relationships within the family leaves us with reservations regarding this placement. The highly disparate known geographic distributions of *Eobogidiella venkataramani* sp. n. and *Eobogidiella purmamarcensis* (India and Argentina, respectively) is suspicious, suggesting that some of their shared character states may be homoplasious. Furthermore, two important morphological characters may be misleading in their support of a close relationship between *Eobogidiella venkataramani* sp. n. and *Eobogidiella purmamarcensis*. First, the soft suture between the head and pereonite 1 described here for *Eobogidiella venkataramani* sp. n. was not mentioned in the description of *Eobogidiella purmamarcensis* (Grosso and Ringuelet 1979) nor in subsequent works treating the placement of this species (Karaman 1981, 1982; Stock 1981; Koenemann and Holsinger 1999). It is likely that the soft suture in *Eobogidiella venkataramani* sp. n. is an artifact caused by the inflation of soft tissues from rehydration of the desiccated specimen. Second, we have described sternal humps as present on pereonites 2–7 of *Eobogidiella venkataramani* sp. n., and these are not mentioned in the description of *Eobogidiella purmamarcensis* nor in subsequent works treating the placement of this species (Karaman 1981, 1982; Stock 1981; Koenemann and Holsinger 1999). Koenemann and Holsinger (1999) included the sternal humps (as “mediosternal processes”) as a character in their phylogenetic analysis, but determined that the mediosternal gills of *Paracrangonyx* evolved independently, coding the mediosternal processes as absent in *Paracrangonyx*. However, the use of sternal humps as a character in the Bogidiellidae did not come into play until well after the treatments of *Eobogidiella purmamarcensis* by Karaman (1981, 1982) and Stock (1981), so the character could have been overlooked. Additionally, we suspect that the occurrence of sternal humps (or “mediosternal processes”) in *Eobogidiella venkataramani* sp. n. is likely independent and does not reflect phylogenetic proximity to the Chilean *Patagongidiella* and *Grossogidiella* (Pérez-Schultheiss 2013).

Based on our reanalysis of Koenemann and Holsinger’s (1999) dataset, relationships among and within genera of the family Bogidiellidae remain unclear. Because the available morphological characters are phylogenetically uninformative, development of additional morphological characters across the family, and, especially, implementation of modern molecular phylogenetic approaches, are desperately needed to resolve relationships within the family and to better define generic boundaries. Nevertheless, it seems that the current assignment of the genera, mostly developed by Koenemann and Holsinger (1999), should be maintained until a more robust and well supported phylogeny can be produced.

## Supplementary Material

XML Treatment for Eobogidiella

XML Treatment for Eobogidiella venkataramani

## Supplementary materials

Supplementary material 1 Morphological character matrix Data type: NEXUS file Explanation note: NEXUS file including character matrix for Bogidiellidae, Artesiidae and Kergueleniolidae used in analysis.. This dataset is made available under the Open Database License (http://opendatacommons.org/licenses/odbl/1.0/). The Open Database License (ODbL) is a license agreement intended to allow users to freely share, modify, and use this Dataset while maintaining this same freedom for others, provided that the original source and author(s) are credited.Dmitry A. Sidorov, Aron D. Katz, Steven J. Taylor, Mikhail V. Chertoprud

Supplementary material 2 Figure S1 Data type: TIF file Explanation note: Ordered bootstrap and jackknife consensus tree. Numbers below branches are bootstrap followed by jackknife support values. This dataset is made available under the Open Database License (http://opendatacommons.org/licenses/odbl/1.0/). The Open Database License (ODbL) is a license agreement intended to allow users to freely share, modify, and use this Dataset while maintaining this same freedom for others, provided that the original source and author(s) are credited.Dmitry A. Sidorov, Aron D. Katz, Steven J. Taylor, Mikhail V. Chertoprud

Supplementary material 3 Figure S2 Data type: TIF file Explanation note: Unordered bootstrap and jackknife consensus tree. Numbers below branches are bootstrap followed by jackknife support values. This dataset is made available under the Open Database License (http://opendatacommons.org/licenses/odbl/1.0/). The Open Database License (ODbL) is a license agreement intended to allow users to freely share, modify, and use this Dataset while maintaining this same freedom for others, provided that the original source and author(s) are credited.Dmitry A. Sidorov, Aron D. Katz, Steven J. Taylor, Mikhail V. Chertoprud
