# Supplementary figures and images for: A reassessment of the phylogenetic utility of genus-level morphological characters in the family Bogidiellidae (Crustacea, Amphipoda), with description of a new species of Eobogidiella Karaman, 1981
Source: Zookeys. 2016 Aug 11;(610):23–43. doi: 10.3897/zookeys.610.9100 (PMC4992811; doi:10.3897/zookeys.610.9100)

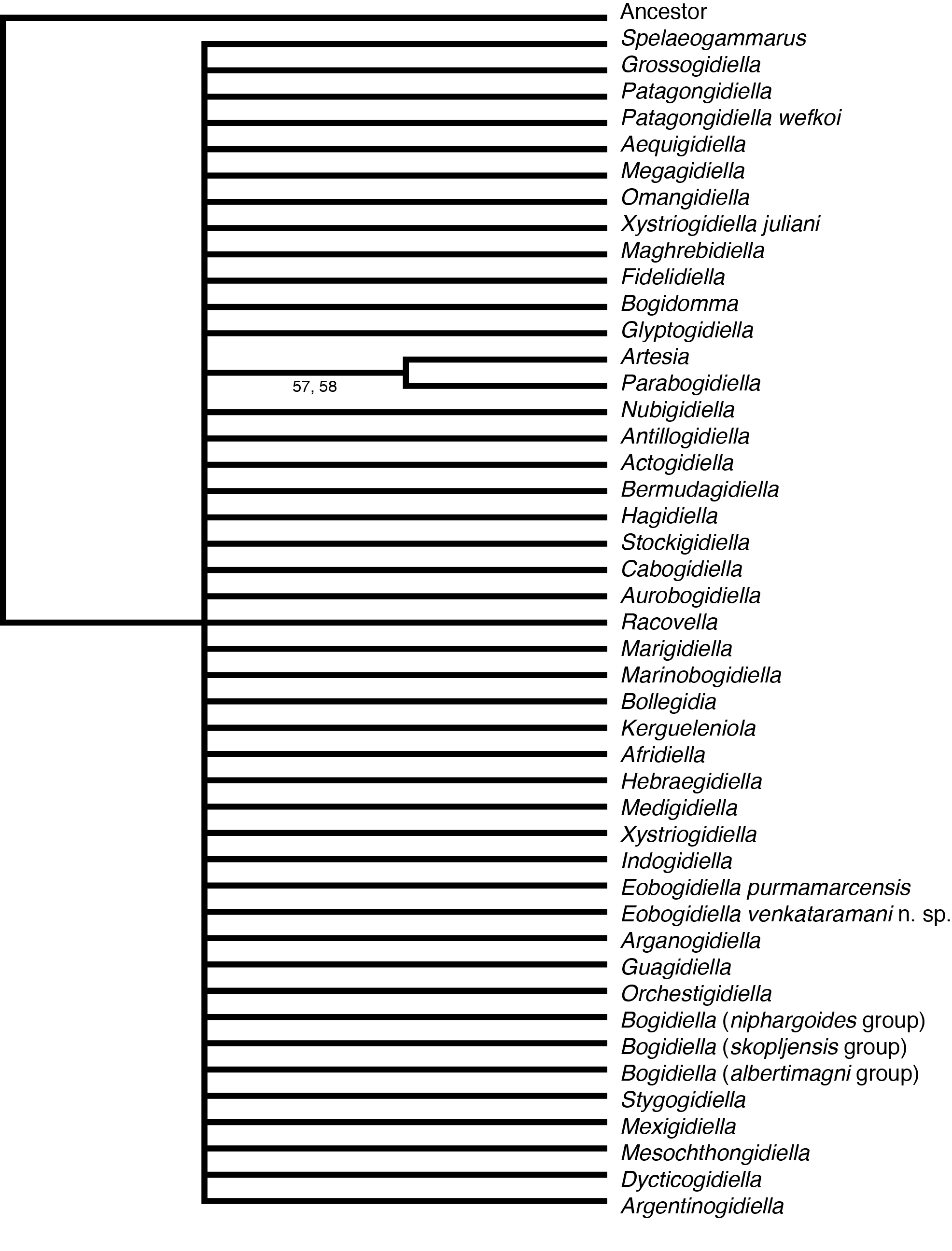

Supplement: Supplementary material 2 — Figure S1 [file zookeys-610-023-s002.tif]

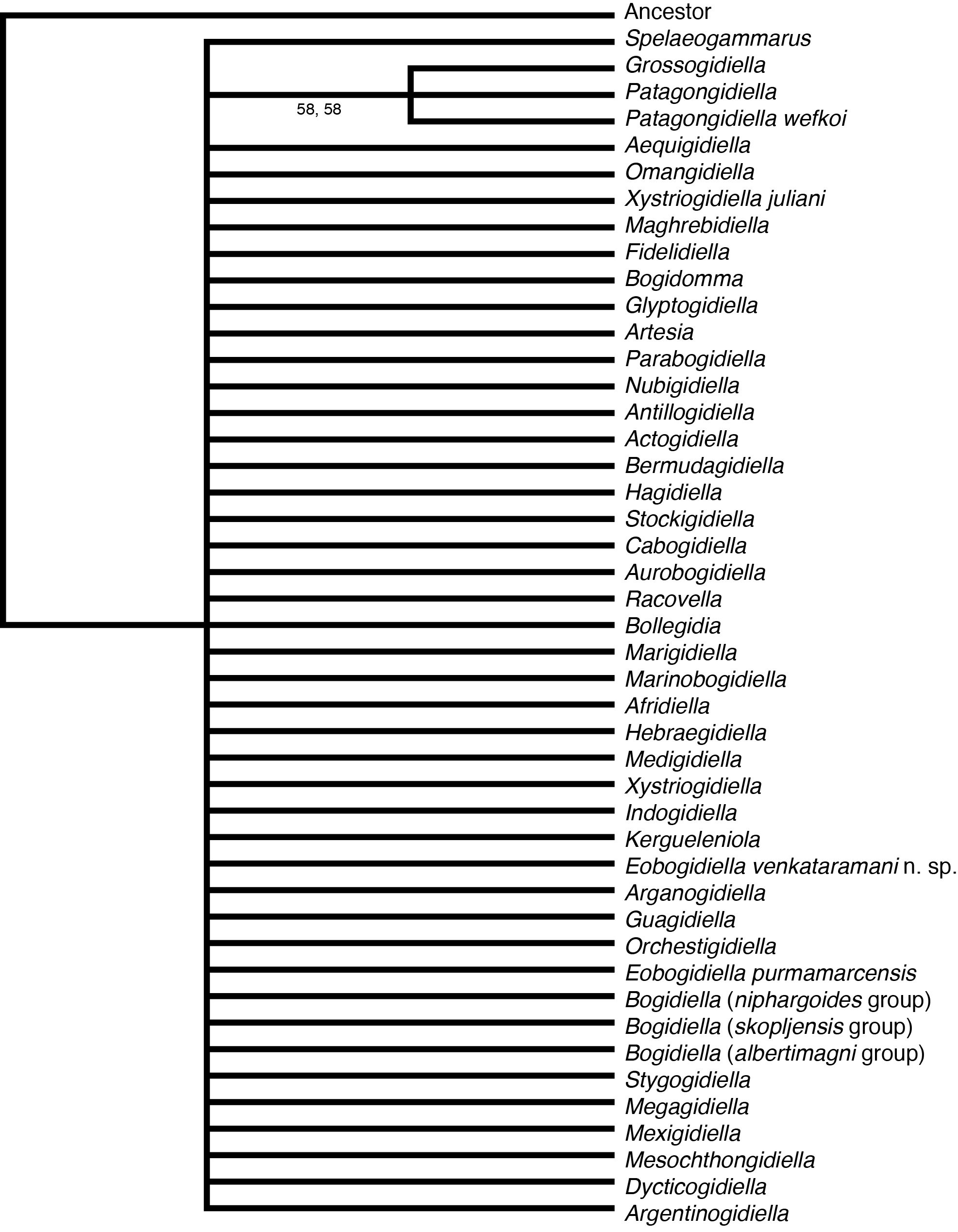

Supplement: Supplementary material 3 — Figure S2 [file zookeys-610-023-s003.tif]
